# Cytopathology of Thoracic SMARCA4-Deficient Undifferentiated Tumor: A Case Report

**DOI:** 10.7759/cureus.82798

**Published:** 2025-04-22

**Authors:** Thomas Harrington, Katsiaryna Khatskevich, Jessica A Forcucci, Jack Yang, Travis Ferguson, Hao Liu

**Affiliations:** 1 Pathology and Laboratory Medicine, Medical University of South Carolina, Charleston, USA; 2 Pulmonary and Critical Care, Medical University of South Carolina, Charleston, USA

**Keywords:** brahma-related gene 1, cytology, diagnostic cytology, thoracic malignancy, thoracic smarca4-deficient undifferentiated tumor

## Abstract

Thoracic SMARCA4-deficient undifferentiated tumor (SMARCA4-UT) is an aggressive tumor with a dismal prognosis, most commonly involving the mediastinum and lungs. The diagnosis of SMARCA4-UT is still challenging, both clinically and pathologically, due to its non-specific clinical findings, a broad list of differential diagnoses, and rarity of cases reported in the literature, especially in terms of cytopathology. Most patients present at an advanced stage of disease at the time of diagnosis, and fine needle aspiration (FNA) cytology specimens and/or core biopsies are often the only diagnostic material available. SMARCA4-UT tumors are strongly associated with a smoking history; however, approximately 10% of the patients are never smokers. We report a case of a 78-year-old male with a remote minimal smoking history who presented with a large mediastinal mass and hilar lymphadenopathy. FNA of the mass demonstrated a moderately cellular aspirate with two populations of loosely cohesive malignant cells in a background of necro-inflammatory debris and bare nuclei. The majority of malignant cells showed enlarged nuclei with prominent nucleoli and scant cytoplasm, with a few malignant cells showing rhabdoid features. Immunohistochemical staining of the cell block showed the malignant cells were patchy positive for INSM-1 and PAX-8; however, negative for keratin markers, TTF-1, Napsin A, and p40. SMARCA4-UT was diagnosed with the loss of SMARCA4 (BRG1) expression in the tumor cells.

## Introduction

Thoracic SMARCA4-deficient undifferentiated tumor (SMARCA4-UT) is a high-grade malignant neoplasm characterized in the 5th edition of the World Health Organization classification of thoracic tumors by an undifferentiated or rhabdoid morphology and characteristic loss of SMARCA4 expression in tumor cells [1,2]. SMARCA4-UT has a male predilection and a strong association with smoking. Tumors frequently have a high mutational burden in smoking-related oncogenic pathways with frequent TP53 mutations. Mutations in SMARCA4 have been shown to co-exist with KRAS mutations but are mutually exclusive from common targetable non-small cell lung carcinoma (NSCLC) oncogenes, including EGFR, ALK, MET, ROS-1, and RET [1,3].

The mammalian switch/sucrose nonfermenting (SWI/SNF) complex is a conserved ATP-dependent chromatin remodeling complex usually ubiquitously expressed in the nuclei of all normal cells as a potent tumor suppressor [1-2,4,5]. Mutations and aberrant expression of some core subunits of the SWI/SNF complex have been associated with tumorigenesis in many malignancies, especially SMARCB1 (SWI/SNF-related matrix-associated actin-dependent regulator of chromatin subfamily B, member 1), SMARCA4, and SMARCA2-deficient tumors [4-6]. SMARCA4 and its encoded protein, Brahma-related gene 1 (BRG1), comprise one member of the conserved SWI/SNF subfamily involved in DNA-histone chromatin remodeling.

Cytology plays an important role in the multimodal workup of SMARCA4-UT, with fine needle aspiration (FNA) tissue often serving as the basis for diagnosis [7-9]. Proper identification of SMARCA4-UT is important due to the propensity for advanced-stage disease presentation with metastasis, necessitating appropriate diagnosis and rapid initiation of appropriate chemoradiation regimens [9].

We present a case of a 78-year-old male with a remote minimal smoking history who presented with a large mediastinal mass and lymphadenopathy determined to be SMARCA4-UT after a multimodal workup of an FNA specimen, including extensive cytomorphological and immunocytochemical characterization. Next-generation sequencing (NGS) was performed with the cell block material.

## Case presentation

**Figure 1:**
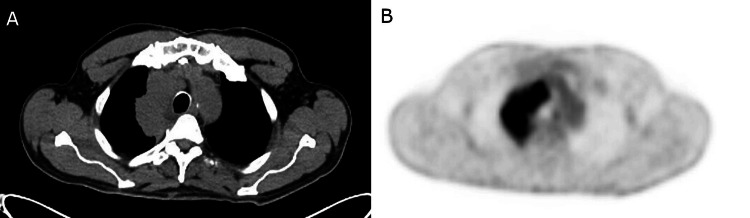
Radiology findings. (A) Chest CT: Right suprahilar/mediastinal conglomerate mass measuring approximately 8.1 x 4.9 x 9 cm. (B) PET scan: Large right hypermetabolic paramediastinal/hilar pulmonary mass concerning for malignancy.

A 78-year-old male presented to the emergency department with a two-week history of chest pain and shortness of breath. He had previously been in good health with no known history of malignancy. The patient had a remote five pack-year smoking history that was reportedly stopped 58 years prior. He reported occupational exposure to smoke from his work as a firefighter. Physical examination revealed bilateral cervical lymphadenopathy. Due to concern for a pulmonary malignancy, a chest CT scan was ordered, which showed a 9 x 8.1 x 4.9 cm conglomerate mass in the right suprahilar and mediastinal region with mild right upper lobe (RUL) bronchial narrowing and partial effacement of the right upper lobe artery. In addition, a small right peri-fissural nodule, right basilar pleural-based nodules, and a peripheral left lower lobe nodule were also noted, concerning for malignancy. The positron emission tomography (PET)-CT scan showed a 9.3 cm thoracic hypermetabolic mass with associated hypermetabolic mediastinal and right supraclavicular lymphadenopathy (Figure 1). Endobronchial ultrasound-guided FNA biopsy of the right mediastinal mass was performed.

**Figure 2:**
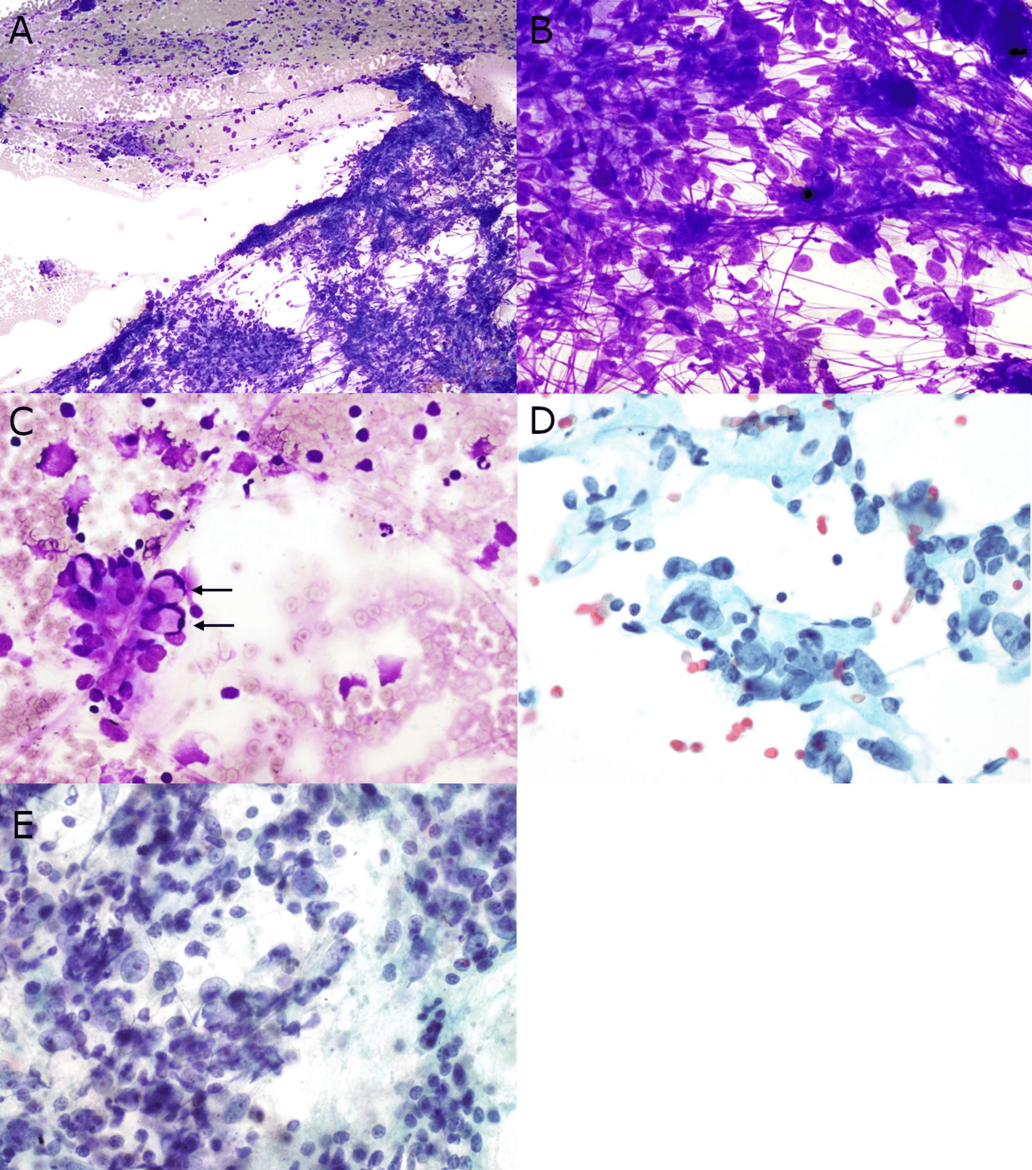
Mediastinal mass fine needle aspirate smear. (A) The aspirate smear shows a moderately cellular specimen with loosely cohesive malignant cells in a background of focal necro-inflammatory debris (Diff-Quik stain, 100x). (B) The majority of the malignant cells show enlarged round to oval nuclei, moderate nuclear polymorphism, prominent nucleoli, and scant cytoplasm with streaking artifact (Diff-Quik stain, 400x). (C and D) Rare malignant cells exhibiting rhabdoid features (arrows) (Diff-Quik stain, 400x and 600x). (E) Papanicolaou-stained smear showed dispersed malignant cells with enlarged nuclei, vesicular chromatin, and prominent nucleoli (600x).

The aspirate smear of the right mediastinal mass showed a moderately cellular specimen comprised of dispersed and loosely cohesive malignant cells in a background of focal necro-inflammatory debris, bare nuclei, and streaking artifact. The majority of malignant cells had enlarged round to oval nuclei with moderate nuclear polymorphism, prominent nucleoli, and scant cytoplasm. A few malignant cells exhibited rhabdoid features. Papanicolaou-stained smear revealed dispersed malignant cells with enlarged nuclei, vesicular chromatin, and prominent nucleoli (Figure 2). The cell block showed malignant cells with similar features. Level 7 lymph node was also biopsied at this time, showing similar appearing malignant cells in a background of lymphocytes. Due to the relatively nonspecific clinical presentation and pathologic findings, and considering the tumor site, the initial differential diagnosis was broad and included poorly differentiated NSCLC, thymic neoplasms, germ cell tumors, lymphoma, melanoma, sarcoma, and metastasis.

**Figure 3:**
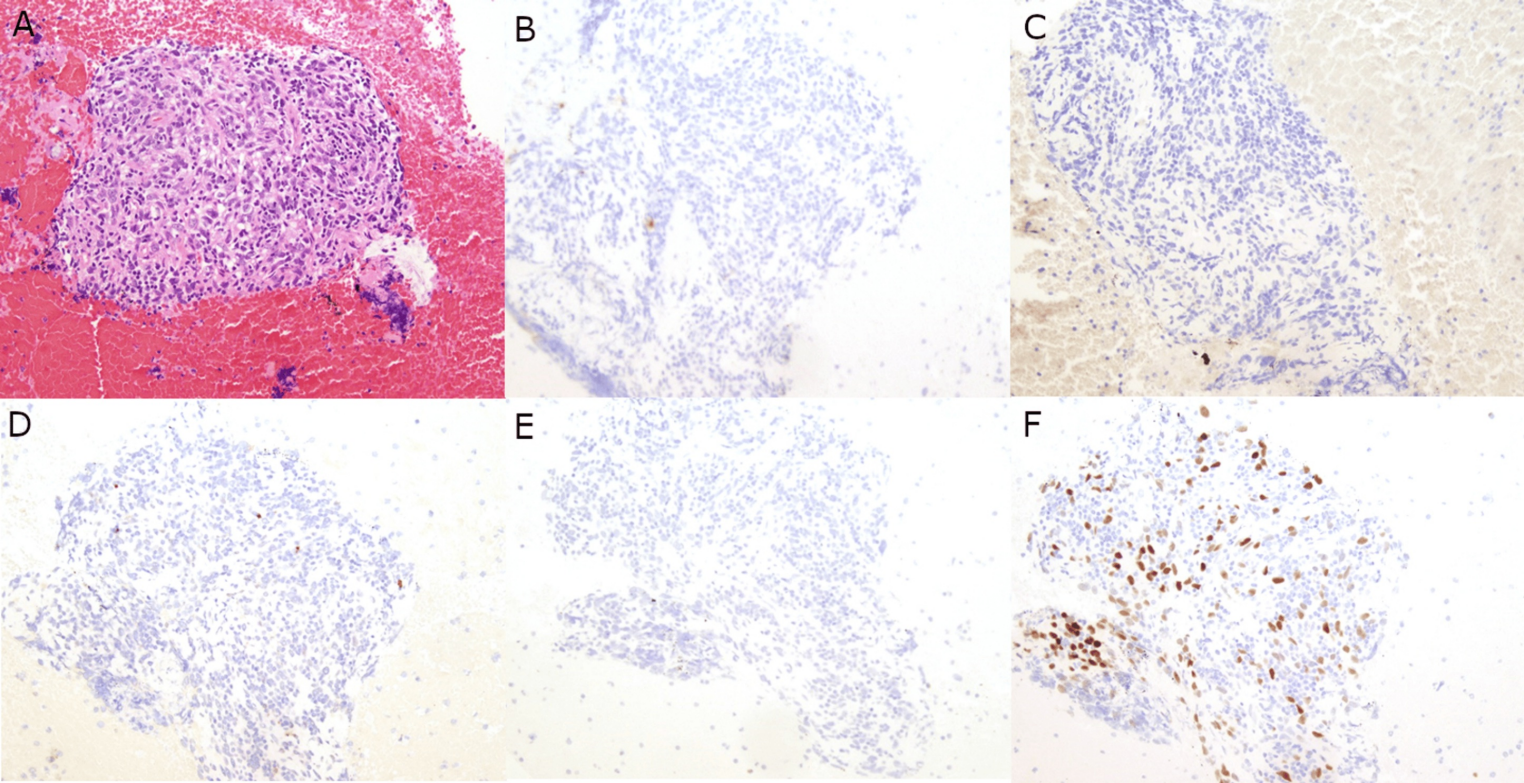
Immunohistochemical profile of the mediastinal mass. (A) The cell block hematoxylin & eosin (H&E) shows malignant cells with enlarged, irregular nuclei, vesicular chromatin, prominent nucleoli, and scant to moderate amounts of cytoplasm present in clusters. The malignant cells are negative for AE1/AE3 (B), Cam5.2 (C), TTF-1/Napsin A dual stain (D), and p40 (E). The malignant cells are patchy positive for INSM-1 (F). Magnification: 200x (A-F).

**Figure 4:**
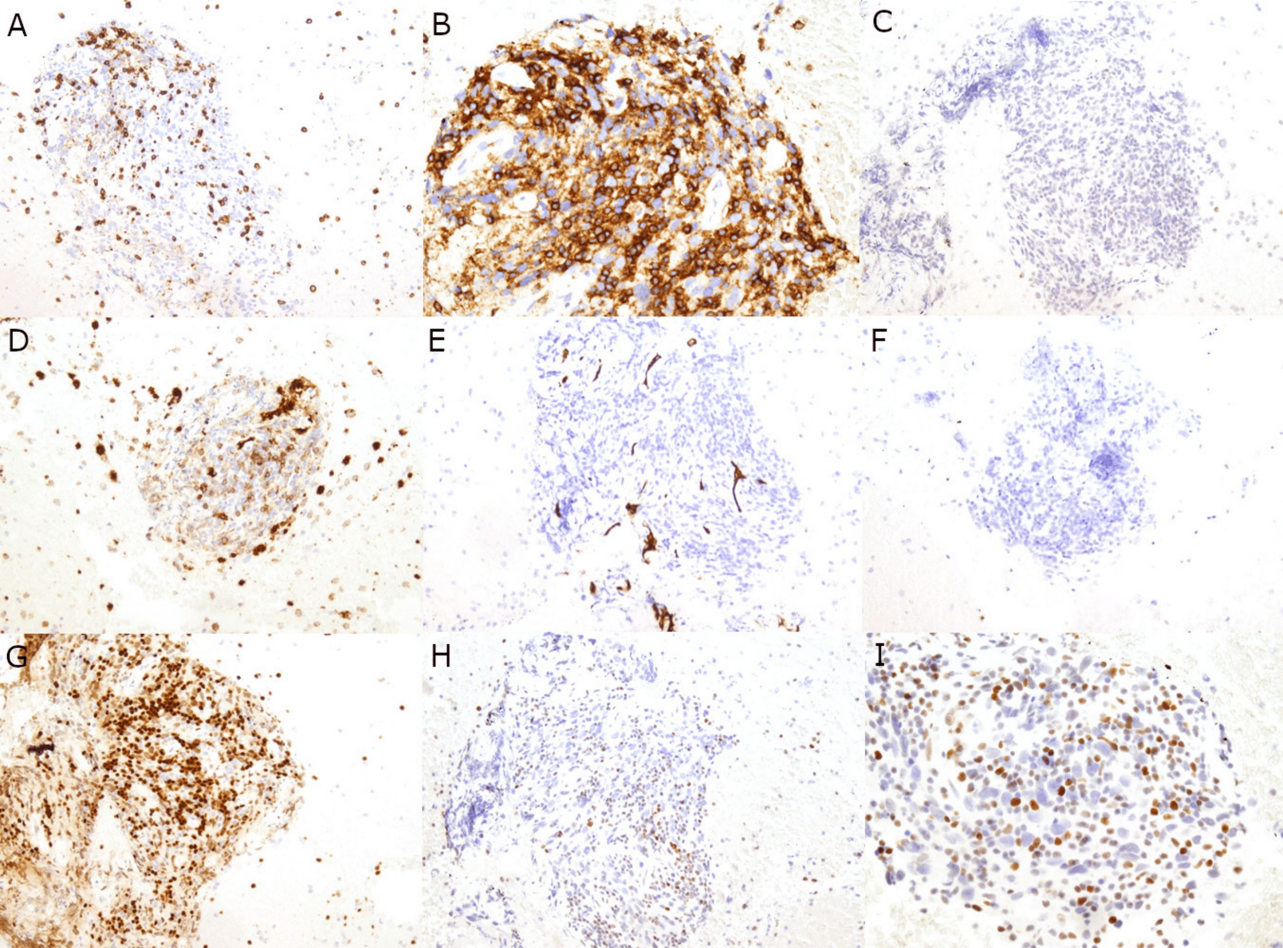
Additional immunohistochemical profile of the mediastinal mass. The malignant cells are negative for CD3 (A), CD20 (B), CD30 (C), CD31 (D), CD34 (E), and OCT3/4 (F). Malignant cells are patchy positive for PAX-8 (G). BRG1 expression is lost in the malignant cells, with only scattered background inflammatory cells staining positive (H and I). Magnification: 200x (A-H) and 400x (I).

Multiple immunohistochemical (IHC) stains were performed on the cell block. The malignant cells were negative for AE1/AE3, Cam5.2, TTF-1, Napsin A, p40, CD3, CD20, CD30, CD45, PAX5, CD31, CD34, OCT3/4, and ALK-1; militating against germ cell tumors, lymphoma, and some sarcomas. Neuroendocrine marker INSM-1 showed patchy positivity in the malignant cells. Interestingly, the malignant cells also showed patchy positivity for PAX-8, and TDT highlighted a few immature T cells, raising the possibility of a thymic neoplasm; however, IHC staining with BRG1 showed malignant cells with loss of SMARCA4/BRG1 expression, rendering the final diagnosis of a SMARCA4-UT (Figures 3, 4). NGS of 49 key cancer-related genes (AKT1, ALK, APC, ARAF, BRAF, CDKN2A, CTNNB1, CYSLTR2, DDR2, EGFR, EIF1AX, ERBB2, ERBB4, ESR1, FBXW7, FGFR1, FGFR2, FGFR3, GNA11, GNAQ, GNAS, H3F3A, HIST1H3B, HRAS, IDH1, IDH2, KEAP1, KIT, KRAS, MAP2K1, MET, NOTCH1, NRAS, NTRK1, PDGFRA, PIK3CA, PLCB4, POLD1, POLE, PTEN, RAC1, RAF1, RET, RNF43, ROS1, SMAD4, STK11, TERT, TP53) was only remarkable for a variant of uncertain significance (VUS) of PIK3CA (P377R). The patient received one cycle of carboplatin and paclitaxel as well as radiation; however, he passed away approximately four months after initial presentation.

## Discussion

SMARCA4-UT is a high-grade tumor that occurs in adults with a wide age range and shows a striking male predominance [1,2]. Clinical symptoms are often nonspecific, such as dyspnea, chest pain, and cough. Radiology usually shows a large, hypermetabolic, ill-defined mass involving the mediastinum, pulmonary hilum, and/or lung with frequent compression of adjacent structures [1,10]. Most patients present in advanced stages of the disease and are not surgical candidates, making FNA cytology and/or core biopsy specimens the only diagnostic material available. Along with limited tissue availability, the diagnosis of SMARCA4-UT can be very challenging to make due to nonspecific clinical findings and broad differential diagnoses. To promote appropriate ancillary tests, a pathologist needs to be familiar with the cytopathological features of SMARCA4-UT; however, only a limited number of cases have been reported.

The cytomorphologic features and IHC profile of our case were consistent with other reported cases of SMARCA4-UT: (1) moderately cellular aspirate containing dispersed and loosely cohesive malignant cells; (2) malignant cells showing nuclear polymorphism, prominent nucleoli, and scant cytoplasm, with occasional cells exhibiting rhabdoid features; (3) a background of necro-inflammatory debris; (4) the tumor cells were focally highlighted by neuroendocrine markers such as INSM-1; (5) loss of expression of SMARCA4 (BRG1) by IHC. Loss of SMARCA4/BRG1 expression plays a vital role in the diagnosis of SMARCA4-UT; however, it is important to note that approximately 5% of conventional NSCLC may also demonstrate deficiency in SMARCA4 and some SMARCA4-UT cases show a severe reduction of SMARCA4/BRG1 expression instead of a complete loss [1]. Unlike SMARCA4-UT, NSCLC with SMARCA4 deficiency usually shows cohesive fragments of malignant cells with at least focal adenocarcinoma or squamous cell differentiation, which can be confirmed with TTF-1, p63/p40, or keratin IHC stains. Expression of SMARCA2 (BRM) is also typically lost in SMARCA4-UT but retained in NSCLC with SMARCA4 deficiency [1,11,12].

The majority of SMARCA4-UT tumors affect heavy smokers and harbor a genomic smoking signature; however, approximately 10% of patients have no smoking history and may present with a different genomic profile [1,2]. This is seen in our case, where our patient lacked a significant cigarette smoking history and the only detectable mutation was PIK3CA P377R, which is a variant of unknown significance. The case lacked a detectable mutation in the common smoking-related carcinogenesis pathway drivers KRAS, STK11, and KEAP1, which are often found in SMARCA4-UT, or TP53, which is commonly co-deleted in these tumors. It should be noted that while the patient reported a minimal cigarette smoking history, he does have a history of working as a firefighter. The significance of his occupational smoke exposure on this disease process is unclear.

## Conclusions

In conclusion, SMARCA4-UT is a rare, high-grade malignancy that often presents without distinct clinical, radiologic, or pathologic features, making the diagnosis of this tumor a challenge. While initial evaluation of the cytology slides may be nonspecific, the use of the BRG1 IHC stain and subsequent molecular workup is essential in arriving at this diagnosis. In addition, the availability of diagnostic tissue is also a frequent problem with these tumors, as most patients present with advanced-stage disease and are inoperable. Awareness of this entity and its cytopathologic features is crucial for pathologists to be able to create an appropriate differential diagnosis, order pertinent IHC stains and molecular workup, and arrive at the correct diagnosis for patients to receive proper treatment.
